# Salvage High-Dose-Rate Brachytherapy for Localized Prostate Cancer Recurrence: A Comparative Analysis of 2 Fractionation Schedules—Single-Institution Study of 223 Patients

**DOI:** 10.1016/j.adro.2026.102210

**Published:** 2026-09-17

**Authors:** Magdalena Stankiewicz, Agata Wilk, Marcela Krzempek, Sylwia Kellas-Ślęczka, Marta Szlag, Piotr Wojcieszek

**Affiliations:** a Brachytherapy Department, Maria Sklodowska-Curie National Research Institute of Oncology, Gliwice Branch, Gliwice, Poland; b Department of Biostatistics and Bioinformatics, Maria Sklodowska-Curie National Research Institute of Oncology, Gliwice Branch, Gliwice, Poland; c Department of Systems Biology and Engineering, Silesian University of Technology, Gliwice, Poland; d Radiotherapy Planning Department, Maria Sklodowska-Curie National Research Institute of Oncology, Gliwice Branch, Gliwice, Poland

## Abstract

**Purpose:**

Salvage high-dose-rate brachytherapy (sHDR-BT) is an effective option for radiorecurrent prostate cancer. This study aimed to compare the efficacy and toxicity of two sHDR-BT fractionation schedules and identify factors associated with treatment outcomes.

**Methods and Materials:**

We retrospectively analyzed 223 patients treated with sHDR-BT between 2008 and 2021. Patients received either 3 × 10 Gy (n = 150) or 2 × 13 Gy (n = 73). Median follow-up was significantly longer in the 3 × 10 Gy group (125 vs 79 months, *P* < .001). Apart from a higher frequency of high-grade disease (*P* = .02) and nodal/distant recurrence (*P* < .001) in the 2 × 13 Gy cohort, baseline characteristics were comparable between the groups. The Kaplan-Meier estimator, log-rank test, and Cox proportional hazards models were used in statistical analysis.

**Results:**

In the entire cohort, the 5-year overall survival rate exceeded 75%, and the median biochemical control was 12.6 years. Factors independently associated with worse oncological outcomes included primary International Society of Urological Pathology grade 5, nodal or distant recurrence, castration resistance, and shorter interval from primary therapy to recurrence. Use of androgen deprivation therapy (ADT) reduced the risk of local failure (*P* = .04). Late genitourinary and gastrointestinal toxicity was acceptable and comparable between the subgroups (*P* = .6 and *P* = .4, respectively). In the no-ADT subgroup (n = 83), median overall survival was 113 months, and median ADT-free survival reached 88 months.

**Conclusions:**

Regardless of the fractionation schedule, sHDR-BT is an effective option for selected patients with localized, noncastration-resistant recurrence, characterized by relatively low toxicity. Castration resistance and regional or distant metastases strongly limit efficacy. In appropriately selected patients, salvage therapy can substantially delay the initiation of ADT.

## Introduction

Prostate cancer (PCa) is the second most commonly diagnosed cancer in men worldwide.^1^ Primary treatment options include radiation therapy (RT), radical prostatectomy (RP), or active surveillance, depending on the disease stage.^2^^,^^3^ In patients with localized PCa, despite dose-escalated RT to the prostate improving biochemical control (BC)^4^^,^^5^ and achieving higher rates of local tumor clearance,^6^ the most common site of first recurrence remains the prostate.^7^ Biochemical and clinical recurrence occurs in approximately 30% to 47% of patients after primary irradiation.^8^ Advances in functional imaging, such as prostate-specific membrane antigen–positron emission tomography/computed tomography and multiparametric magnetic resonance imaging, have revealed that locally persistent disease may be more prevalent than previously appreciated.^9^, ^10^, ^11^

The main challenge in managing recurrent PCa is to identify a personalized treatment strategy that prevents metastatic progression or symptomatic local progression although minimizing adverse effects and preserving quality of life (QoL).^12^ The optimal therapeutic approach in this clinical setting remains debated.^12^^,^^13^ According to European and American guidelines, salvage treatment options include androgen deprivation therapy (ADT), RP, brachytherapy (BT), stereotactic ablative RT, high-intensity focused ultrasound, and cryoablation.^2^^,^^3^

In the MASTER meta-analysis, salvage prostatectomy, BT, stereotactic ablative RT, cryotherapy, and high-intensity focused ultrasound demonstrated comparable 5-year BC rates, although toxicity profiles differed substantially. Notably, salvage BT—both high-dose-rate (HDR) and low-dose-rate—was associated with lower rates of severe urinary toxicity compared with salvage prostatectomy, and HDR-BT was associated with lower rates of severe gastrointestinal toxicity compared with other salvage options.^14^ The most robust evidence for salvage BT comes from the Radiation Therapy Oncology Group (RTOG)-0526, a multi-institutional, prospective, single-arm trial of salvage low-dose-rate BT. In this study, no grade 4 or 5 toxicities were observed, whereas 14% of patients experienced late grade 3 toxicity.^15^ Long-term outcomes were favorable, with only 5% experiencing local failure and a 10-year biochemical failure rate of 46%.^16^ RTOG-0526 applied strict eligibility criteria, enrolling only patients with low- or intermediate-risk PCa and prostate-specific antigen (PSA) ≤10 ng/mL at salvage. In clinical practice, however, patient selection tends to be more permissive. The Groupe Européen de Curiethérapie-European Society for Radiotherapy and Oncology consensus underscored disagreements among experts regarding optimal salvage BT approaches.^17^ Similarly, the Delphi consensus by Corkum et al^13^ identified uncertainties, including patient eligibility (eg, maximum T stage, PSA levels, and oligometastases), the role and duration of ADT, and treatment technique, highlighting the need for evidence-based guidance.

Because of the scarcity of prospective data and the heterogeneity of current clinical practice, we conducted a retrospective analysis to evaluate real-world outcomes of salvage HDR-BT in radiorecurrent PCa, aiming to identify the most effective and safe fractionation scheme.

## Methods and Materials

In this retrospective study, we evaluated the efficacy and toxicity of salvage HDR-BT (sHDR-BT) for locally recurrent PCa after primary irradiation. Data were collected for patients treated between April 2008 and January 2021. Eligible patients had a confirmed local recurrence, a minimum follow-up of 6 months after treatment, and received radical salvage therapy with interstitial HDR-BT using 1 of 2 fractionation schedules: (1) 10 Gy per fraction delivered in 3 fractions to a total dose of 30 Gy or (2) 13 Gy per fraction delivered in 2 fractions to a total dose of 26 Gy, which are commonly regarded as radiobiologically comparable high-dose hypofractionated regimens. Prostate volume was not a limiting factor, provided there was no pubic arch interference. Patients with prior RP or treated with other HDR-BT fractionation regimens were excluded from the analysis. Initially, 232 patients were identified. Nine patients were excluded because of missing follow-up data, resulting in a final study cohort of 223 patients. The study protocol was approved by the institutional Ethics Committee (approval No. 54/2023), with informed consent waived because of its retrospective design.

### Primary treatment

Baseline disease characteristics at primary diagnosis were comparable between treatment groups with respect to the National Comprehensive Cancer Network risk group at initial diagnosis, the International Society of Urological Pathology (ISUP) grade distribution, and the maximum PSA level at initial treatment. However, the groups differed significantly in the primary treatment approach, with the 2 × 13 Gy group more frequently having a history of stereotactic body RT or BT alone (27% vs 2%), whereas patients treated with 3 × 10 Gy more commonly underwent external beam RT with a BT boost (27% vs 8%). Elective nodal irradiation was performed in 41% of patients in the 3 × 10 Gy group and in 38% of patients in the 2 × 13 Gy group.

### sHDR-BT procedure

A total of 150 patients received 10 Gy per fraction in 3 fractions over 28 to 30 days, whereas 73 patients received 13 Gy per fraction in 2 fractions over 7 to 14 days. All procedures were performed in the operating theater of the Brachytherapy Department. Implantation was conducted under epidural anesthesia, or general anesthesia when contraindicated, in the lithotomy position, using transrectal ultrasound guidance with either plastic 6F tubes or metal needles.

The clinical target volume (CTV) encompassed the entire prostate. In patients with seminal vesicle involvement, the affected seminal vesicles were also included in the CTV. No separate planning target volume was generated, and treatment planning was based directly on the CTV without additional margins. No image fusion with diagnostic imaging was performed for treatment planning. The dominant intraprostatic lesion was not separately contoured, and no focal dose escalation was applied. The urethra, rectal wall, and urinary bladder (represented by the Foley catheter balloon) were delineated as organs at risk.

The planning objective was to deliver at least 90% of the prescribed dose to 100% of the CTV (V_100_ ≥90%). Detailed dosimetry data were available for 40% of the patients. Recommended and achieved dose-volume parameters are presented in Table E1.

Treatment planning was performed both virtually and in real time using the Oncentra Prostate system (Nucletron, Elekta AB). Brachytherapy was delivered with an iridium-192 (^192^Ir) source (MicroSelectron or Flexitron, Nucletron, Elekta AB). After treatment delivery, all applicators were removed immediately.

Biologically effective doses (BEDs) were calculated using the linear-quadratic model with an α/β ratio of 1.5 Gy. BEDs from the primary treatment and cumulative BEDs for both fractionation regimens are presented in Table E2.

### Follow-up

All patients were followed at our center. Each visit included assessment of genitourinary (GU) and gastrointestinal (GI) toxicity, and PSA measurement. Additional imaging was performed when clinically indicated, for example, in cases of rising PSA, abnormal findings on digital rectal examination, or new clinical symptoms. Biochemical relapse was defined according to the Phoenix criteria (PSA nadir + 2 ng/mL).^18^ Acute and late toxicities were graded using the National Cancer Institute Common Terminology Criteria for Adverse Events, version 5.0.^19^ Sexual function was not assessed in this study.

### Study objectives

The primary endpoint was local control (LC), defined as the interval from completion of sHDR-BT to histopathologically or radiologically confirmed local recurrence. Secondary endpoints were BC, defined by the Phoenix criteria, distant metastasis–free survival (DMFS), disease-free survival (DFS), and overall survival (OS). All time-to-event endpoints were calculated from the date of the last sHDR-BT fraction and were censored at the last follow-up for patients without the event. Treatment safety was evaluated according to the National Cancer Institute Common Terminology Criteria for Adverse Events, version 5.0. Time to adverse event was measured from the first day of sHDR-BT, with acute toxicity defined as ≤180 days and late toxicity as >180 days posttreatment. The following variables were analyzed as potential predictors of survival outcomes: age at recurrence, maximum PSA at recurrence, ISUP grade at primary diagnosis and recurrence, ADT use at recurrence, disease stage at recurrence, primary treatment modality, National Comprehensive Cancer Network risk group at initial diagnosis, time to recurrence after primary treatment, sHDR-BT fractionation schedule, and presence of castration-resistant PCa (CRPC) at recurrence (including patients meeting CRPC criteria based on PSA kinetics with testosterone levels not determined).

### Statistical analysis

Descriptive statistics were used to summarize baseline characteristics and treatment modalities. Follow-up was estimated using the reverse Kaplan-Meier method. Group comparisons for baseline characteristics were performed using the Mann-Whitney *U* test for continuous variables and the Fisher exact test for categorical variables. Survival analysis was performed using the Kaplan-Meier estimator, with the log-rank test for group comparisons. Cox proportional hazards regression models were applied to identify predictors of progression and survival, adjusting for relevant covariates. Only patients with complete information for the considered variables were included in the analysis (n = 223 for DFS, LC, and DMFS; and n = 209 for BC). For each endpoint, 3 approaches were evaluated: univariable analysis for each variable, a full model containing all variables, and a reduced model obtained by stepwise elimination based on the Akaike information criterion. The analysis was repeated for a subgroup of patients with localized disease only (N0M0; n = 195 for DFS, LC, and DMFS; and n = 182 for BC). Toxicity rates were reported as frequencies and percentages, categorized by severity. The toxicity comparison between fractionation schemes was performed using the 2-sided Fisher exact test for count data, and Cramér V was used to estimate the effect size. Hazard ratios (HRs) are reported with 95% CIs, and *P* <.05 was considered statistically significant. All analyses were performed using Stata (StataCorp, 2025. StataNow 19.5 [computer software]; StataCorp LLC; available from https://www.stata.com), Statistica 13.1 (StatSoft Incorporated), and the R environment for statistical computing (version 4.1.3, R Foundation for Statistical Computing; http://www.r-project.org).

## Results

### Patients characteristics

A total of 223 patients met the inclusion criteria and were included in this retrospective analysis: 150 received 3 × 10 Gy and 73 received 2 × 13 Gy. Data were obtained from a retrospective chart review. Median follow-up was significantly longer in the 3 × 10 Gy group (125 vs 79 months; *P* < .001). Patients treated with 2 × 13 Gy more frequently presented with higher-grade disease (ISUP G4-G5: 23% vs 13%) and nodal or distant metastases (N1 or M1: 36% vs 1%). At initial diagnosis, they were less likely to have received external beam RT with a BT boost (8% vs 27%). No significant differences were observed between groups in PSA at recurrence, use of neoadjuvant ADT, time to recurrence, or incidence of castration-resistant disease. Detailed patient characteristics are presented in Table 1. A small number of patients with oligometastatic (M1) recurrence were included after multidisciplinary discussion. In these cases, owing to the absence of other potentially curative treatment options, sHDR-BT to the local recurrence was combined with stereotactic body RT to all metastatic lesions.

**Table 1:** Baseline patient characteristics.

			Subgroup according to fractionation regimen		
Characteristic	Measure	Overall cohort (n = 223)	3 × 10 Gy (n = 150)	2 × 13 Gy (n = 73)	*P* value
Age at sHDR-BT (y)	Median (Q1-Q3)	71 (66-74)	70 (66-74)	72 (67-75)	.09
Follow-up time (mo)	Median (Q1-Q3)	92 (51-137)	125 (92-144)	79 (73-87)	<.001⁎
PSA at recurrence (ng/mL)	Median (Q1-Q3)	3.3 (2.1-5.4)	3.1 (1.9-5.1)	3.6 (2.7-5.4)	.1
ISUP grade at recurrence	n (%)				
G1		33 (14.8)	25 (16.7)	8 (11.0)	.02
G2		38 (17.0)	27 (18.0)	11 (15.1)	
G3		22 (9.9)	9 (6.0)	13 (17.8)	
G4		29 (13.0)	15 (10.0)	14 (19.2)	
G5		8 (3.6)	5 (3.3)	3 (4.1)	
NA		93 (41.7)	69 (46.0)	24 (32.9)	
Disease stage at recurrence	n (%)				
LR only		195 (87.4)	148 (98.7)	47 (64.4)	<.001
LR N1 only		14 (6.3)	1 (0.7)	13 (17.8)	
LR M1		14 (6.3)	1 (0.7)	13 (17.8)	
Time to recurrence (mo)	Median (Q1-Q3)	72 (50-98)	72 (52-96)	75 (48-105)	.9
Neoadjuvant ADT at recurrence	n (%)				
No		83 (37.2)	60 (40.0)	23 (31.5)	.2
Yes		140 (62.8)	90 (60.0)	50 (68.5)	
ADT duration at recurrence (mo)	Median (Q1-Q3)	25 (12-48)	28 (13-52)	19 (11-40)	.09
Time from ADT initiation to sHDR-BT (mo)	Median (Q1-Q3)	7 (3-26)	8 (4-30)	5 (2-15)	.02
CR† at recurrence	n (%)				
No		201 (90.1)	133 (88.7)	68 (93.2)	.3
Yes		22 (9.9)	17 (11.3)	5 (6.8)	
Disease stage at primary diagnosis‡	n (%)				
Low risk		51 (22.9)	35 (23.3)	16 (21.9)	.3
Intermediate risk		82 (36.8)	59 (39.3)	23 (31.5)	
High risk		89 (39.9)	56 (37.3)	33 (45.2)	
M1		1 (0.4)	-	1 (1.4)	
Primary ISUP grade	n (%)				
G1		117 (52.5)	78 (52.0)	39 (53.4)	.7
G2		44 (19.7)	33 (22.0)	11 (15.1)	
G3		19 (8.5)	12 (8.0)	7 (9.6)	
G4		22 (9.9)	13 (8.7)	9 (12.3)	
G5		6 (2.7)	3 (2.0)	3 (4.1)	
NA		15 (6.7)	11 (7.3)	4 (5.5)	
Primary treatment	n (%)				
EBRT		153 (68.6)	106 (70.7)	47 (64.4)	<.001
EBRT + BT boost		47 (21.1)	41 (27.3)	6 (8.2)	
SBRT		10 (4.5)	1 (0.7)	9 (12.3)	
BT		13 (5.8)	2 (1.3)	11 (15.1)	
Primary PSA max (ng/mL)	Median (Q1-Q3)	13.5 (8.0-29.3)	12.6 (7.8-27.0)	14.0 (8.6-31.6)	.3

*Abbreviations:* ADT = androgen deprivation therapy; BT = brachytherapy; CR = castration resistance; CRPC = castration-resistant prostate cancer; EBRT = external beam radiation therapy; ISUP = International Society of Urological Pathology; LR = local recurrence; NA = not available; PSA = prostate-specific antigen; Q1, Q3 = first and third quartiles; SBRT = stereotactic body radiation therapy; sHDR-BT = salvage high-dose-rate brachytherapy.

⁎ Log-rank test *P* value.

† Including patients with PSA-defined CRPC without testosterone assessment.

‡ According to the National Comprehensive Cancer Network.

### Oncological outcomes

#### Whole group

In the entire cohort of patients treated with salvage BT, LC, and DMFS outcomes were favorable, with median values not reached. Median BC was 151 months, and OS exceeded 75% at 5 years (Table 2). No significant differences were observed between fractionation schedules for any of the oncological outcomes (Fig. 1). On both univariable and multivariable analyses, the fractionation schedule was not significantly associated with BC, LC, DFS, or DMFS (Table E3).

**Table 2:** Oncological outcomes in the whole cohort, local recurrence only (N0M0) subgroup, and no-ADT subgroup.

Patient subgroup	OS	BC	LC	DMFS	DFS
All patients (n = 223)	Median: 108 mo (102-128) 2 y: 96% (93%-98%) 5 y: 76% (70%-82%)	Median: 151 mo (87-NR) 2 y: 74% (69%-80%) 5 y: 62% (56%-69%)	Median: NR 2 y: 93% (90%-97%) 5 y: 79% (74%-85%)	Median: NR 2 y: 86% (82%-91%) 5 y: 75% (69%-81%)	Median: 88 mo (71-137) 2 y: 82% (77%-87%) 5 y: 61% (55%-68%)
N0M0 patients (n = 195)	Median: 108 mo (101-128) 2 y: 95% (92%-98%) 5 y: 76% (70%-82%)	Median: 151 mo (91-NR) 2 y: 75% (69%-81%) 5 y: 63% (57%-70%)	Median: NR 2 y: 94% (90%-97%) 5 y: 82% (76%-88%)	Median: NR 2 y: 88% (84%-93%) 5 y: 77% (71%-83%)	Median: 105 mo (80-NR) 2 y: 84% (79%-89%) 5 y: 65% (58%-72%)
No-ADT group (n = 83)	Median: 113 mo (104-NR) 2 y: 98% (94%-100%) 5 y: 84% (77%-93%)	Median: 151 mo (98-NR) 2 y: 76% (67%-86%) 5 y: 67% (57%-78%)	Median: 137 mo (116-NR) 2 y: 96% (92%-100%) 5 y: 80% (71%-89%)	Median: NR 2 y: 92% (86%-98%) 5 y: 84% (77%-92%)	Median: 116 mo (87-NR) 2 y: 89% (83%-96%) 5 y: 70% (61%-81%)

*Abbreviations:* ADT = androgen deprivation therapy; BC = biochemical control; DFS = disease-free survival; DMFS = distant metastasis–free survival; LC = local control; NR = not reached; OS = overall survival.

95% CIs are given in brackets.

**Figure 1:**
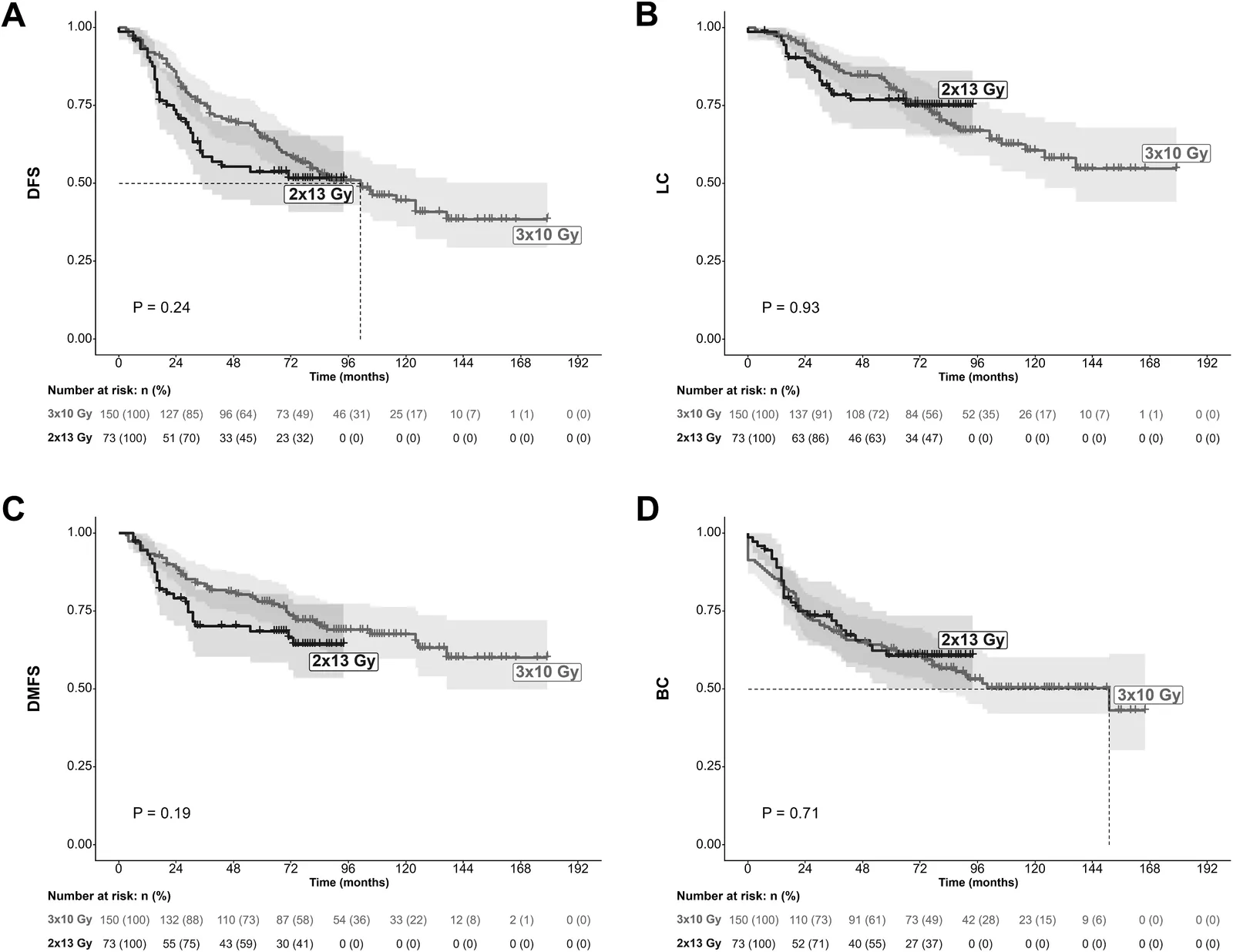
Comparison of oncological outcomes between the 2 fractionation schedules in the whole cohort. (A) DFS. (B) LC. (C) DMFS. (D) BC. *Abbreviations*: BC = biochemical control; DFS = disease-free survival; DMFS = distant metastasis–free survival; LC = local control.

Several independent prognostic factors were identified in multivariable models. Primary ISUP grade 5 (HR, 8.66; *P* < .001), M1 stage at recurrence (HR, 3.90; *P* < .001), higher maximum PSA at recurrence (HR, 1.06; *P* = .02), and castration resistance (CR) at the time of sHDR-BT (HR, 2.31; *P* = .01) were adverse prognostic factors for DFS. Similarly, factors independently linked to worse LC were primary ISUP grade 5 (HR, 11.35; *P* < .001), younger age at salvage treatment (HR, 0.94; *P* = .009), N1 stage at recurrence (HR, 2.74; *P* = .045), CR at sHDR-BT (HR, 3.14; *P* = .006), and a longer interval between recurrence diagnosis and local therapy (HR, 1.03; *P* = .005). Use of ADT was associated with a reduced risk of local failure (HR, 0.47; *P* = .01). Regarding DMFS, adverse prognostic factors were M1 stage at recurrence (HR, 2.87; *P* = .01), higher maximum PSA at recurrence (HR, 1.07; *P* = .006), primary ISUP grade 5 (HR, 7.17; *P* < .001), and CR at recurrence (HR, 3.78; *P* < .001). For BC, worse outcomes were associated with M1 stage at recurrence (HR, 3.74; *P* = .001), higher maximum PSA at recurrence (HR, 1.13; *P* < .001), and shorter time interval from primary treatment to recurrence (HR, 0.99; *P* = 0.001). A summary of the reduced multivariable models (Akaike information criterion) for BC, LC, DMFS, and DFS is provided in Table 3.

**Table 3:** Multivariable analysis—reduced model (AIC criterion) of factors associated with oncological outcomes in the whole cohort.

Outcome	Factor	Category	Event/n	HR (95% CI)	*P* value
DFS (n = 223)	Disease stage at recurrence	N0M0 (Ref)	90/195	-	-
		N1	7/14	1.98 (0.87-4.50)	.1
		M1	11/14	3.90 (1.97-7.71)	<.001
	Age at sHDR-BT	-	108/223	0.97 (0.93-1.00)	.06
	PSA at recurrence	-	108/223	1.06 (1.01-1.11)	.02
	Primary ISUP grade	1 (Ref)	47/117	-	-
		2	26/44	1.82 (1.06-3.13)	.03
		3	9/19	1.02 (0.48-2.18)	.96
		4	15/22	1.18 (0.60-2.34)	.6
		5	6/6	8.66 (3.05-24.60)	<.001
		NA	5/15	0.99 (0.38-2.60)	.99
	Primary NCCN risk group	Low (Ref)	18/51	-	-
		Intermediate	34/82	0.61 (0.31-1.18)	.1
		High/M1	56/90	1.16 (0.60-2.23)	.7
	T stage at recurrence	T1 (Ref)	16/32	-	-
		T2	63/151	0.68 (0.38-1.20)	.2
		T3	29/40	1.67 (0.87-3.21)	.1
	Time from primary treatment to recurrence diagnosis	-	108/223	1.00 (0.99-1.00)	.1
	CR⁎ at recurrence	No (Ref)	90/201	-	-
		Yes	18/22	2.31 (1.20-4.46)	.01
LC (n = 223)	Disease stage at recurrence	N0M0 (Ref)	54/195	-	-
		N1	5/14	2.74 (1.02-7.37)	.045
		M1	5/14	2.29 (0.86-6.11)	.1
	Age at sHDR-BT	-	64/223	0.94 (0.91-0.99)	.009
	Primary ISUP grade	1 (Ref)	26/117	-	-
		2	13/44	0.87 (0.41-1.82)	.7
		3	8/19	2.07 (0.92-4.67)	.08
		4	9/22	1.60 (0.73-3.49)	.2
		5	4/6	11.35 (3.60-35.84)	<.001
		NA	4/15	1.42 (0.48-4.19)	.5
	T stage at recurrence	T1 (Ref)	9/32	-	-
		T2	40/151	0.70 (0.33-1.50)	.4
		T3	15/40	1.38 (0.58-3.27)	.5
	Time from recurrence to sHDR-BT	-	64/223	1.03 (1.01-1.05)	.005
	Use of ADT	No (Ref)	28/83	-	-
		Yes	36/140	0.47 (0.27-0.84)	.01
	CR⁎ at recurrence	No (Ref)	54/201	-	-
		Yes	10/22	3.14 (1.38-7.11)	.006
DMFS (n = 223)	Disease stage at recurrence	N0M0 (Ref)	59/195	-	-
		N1	4/14	1.81 (0.63-5.22)	.3
		M1	7/14	2.87 (1.27-6.48)	.01
	Age at sHDR-BT	-	70/223	0.97 (0.93-1.01)	.1
	Max PSA at recurrence	-	70/223	1.07 (1.02-1.12)	.006
	Primary ISUP grade	1 (Ref)	31/117	-	-
		2	17/44	1.35 (0.73-2.47)	.3
		3	5/19	0.84 (0.32-2.21)	.7
		4	9/22	1.38 (0.63-3.01)	.4
		5	6/6	7.17 (2.56-20.11)	<.001
		NA	2/15	0.55 (0.13-2.38)	.4
	Time from primary treatment to recurrence	-	70/223	0.99 (0.98-1.00)	.09
	CR⁎ at recurrence	No (Ref)	55/201	-	-
		Yes	15/22	3.78 (1.86-7.68)	<.001
BC (n = 209)	Disease stage at recurrence	N0M0 (Ref)	70/182	-	-
		N1	3/14	0.56 (0.17-1.87)	.3
		M1	8/13	3.74 (1.68-8.33)	.001
	Age at sHDR-BT	-	81/209	0.97 (0.94-1.01)	.1
	Max PSA at recurrence	-	81/209	1.13 (1.07-1.18)	<.001
	Primary ISUP grade	1 (Ref)	35/111	-	-
		2	19/39	2.78 (1.41-5.49)	.003
		3	8/18	2.01 (0.85-4.76)	.1
		4	9/20	1.33 (0.58-3.06)	.5
		5	5/6	5.96 (1.87-19.02)	.003
		NA	5/15	2.17 (0.77-6.10)	.1
	Primary NCCN risk group	Low (Ref)	16/49	-	-
		Intermediate	23/76	0.31 (0.14-0.69)	.004
		High/M1	42/84	0.96 (0.47-1.95)	.9
	T stage at recurrence	T1 (Ref)	10/29	-	-
		T2	49/142	0.84 (0.41-1.72)	.6
		T3	22/38	2.12 (0.96-4.66)	.06
	Time from primary treatment to recurrence	-	81/209	0.99 (0.98-1.00)	.002
	Use of ADT	No (Ref)	27/78	-	-
		Yes	54/131	0.63 (0.36-1.08)	.09
	CR⁎ at recurrence	No (Ref)	68/191	-	-
		Yes	13/18	2.17 (0.99-4.79)	.05

*Abbreviations:* ADT = androgen deprivation therapy; AIC = Akaike information criterion; BC = biochemical control; CI - confidence interval; CR = castration resistance; CRPC = castration-resistant prostate cancer; DFS = disease-free survival; DMFS = distant metastasis–free survival; HR = hazard ratio; ISUP = International Society of Urological Pathology; LC = local control; NA = not available; NCCN = National Comprehensive Cancer Network; PSA = prostate-specific antigen; Ref = reference; sHDR-BT = salvage high-dose-rate brachytherapy.

⁎ Including patients with PSA-defined CRPC without testosterone assessment.

#### N0M0 subgroup

Patients with local recurrence alone demonstrated slightly better outcomes compared with the overall cohort (Table 2). In this subgroup, no statistically significant differences in survival outcomes were observed between the 2 fractionation regimens (Fig. 2). In Cox regression analyses, fractionation schedule was not an independent predictor of any oncological endpoint (BC, LC, DFS, or DMFS). In contrast, CR at the time of sHDR-BT consistently correlated with worse outcomes, including DFS (HR, 2.48; *P* = .007), LC (HR, 3.82; *P* = .002), and DMFS (HR, 2.72; *P* = .01). Higher PSA level at recurrence was independently associated with inferior DFS (HR, 1.05; *P* = .03), DMFS (HR, 1.07; *P* = .002), and BC (HR, 1.14; *P* < .001). Full results of these analyses are provided in Table E4.

**Figure 2:**
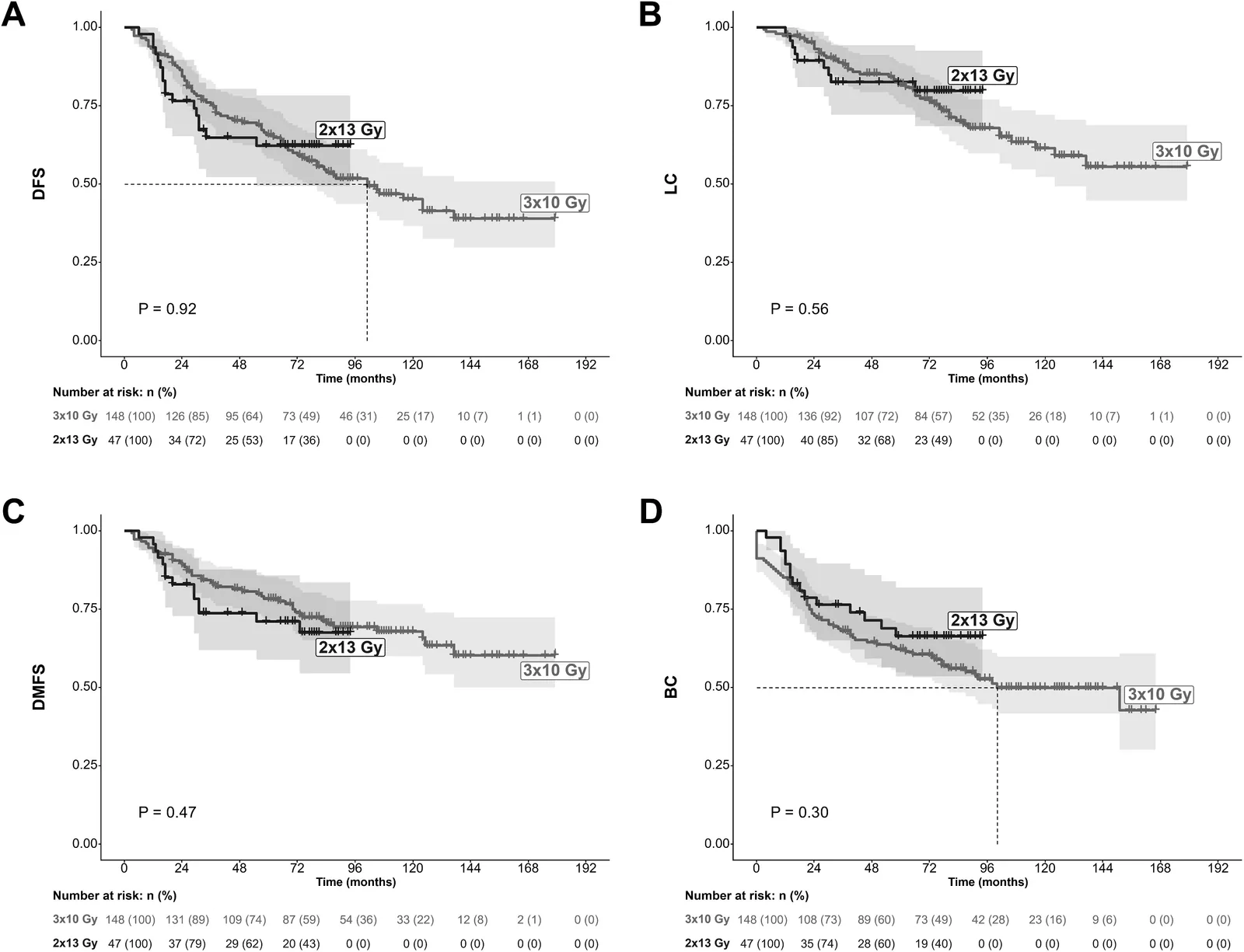
Comparison of oncological outcomes between the 2 fractionation schedules in the subgroup with local recurrence only (N0M0). (A) DFS. (B) LC. (C) DMFS. (D) BC. *Abbreviations*: BC = biochemical control; DFS = disease-free survival; DMFS = distant metastasis–free survival; LC = local control.

#### Subgroup without ADT

In patients who did not receive ADT before, during, or within 1 month after completion of sHDR-BT, OS outcomes were favorable, with a median OS of 113 months and a 5-year OS rate of 84%. Median ADT-free survival was 88 months, and 5-year ADT-free survival was 54%. However, because of the smaller sample size and limited number of events in this subgroup, Cox proportional hazards modeling could not reliably determine prognostic factors for most oncological outcomes. N1 stage at recurrence was identified as an independent adverse prognostic factor, being significantly associated with worse DFS (HR, 19.28; *P* < .001), inferior LC (HR, 9.93; *P* < .001), and shorter ADT-free survival (HR, 17.83; *P* < .001). Full results of these analyses are provided in Table E5.

#### Toxicity

Both fractionation regimens demonstrated acceptable acute and late toxicity profiles. Acute GU toxicity profiles differed significantly (with moderate association) between treatment regimens for the whole cohort (*P* = .007; Cramér V = 0.22), and in the N0M0 subset (*P* = .02; Cramér V = 0.21), with lower toxicity in the 2 × 13 Gy group. In contrast, acute GI toxicity did not significantly differ between fractionation schedules, either in the overall population (*P* = .23) or in the N0M0 subgroup (*P* = .55). Importantly, late GU and GI toxicities were generally low in both regimens, with no statistically significant differences. In the full cohort, late grade 3 GU toxicity occurred in 2.7% of patients treated with 3 × 10 Gy and in 4.1% of those treated with 2 × 13 Gy. Late grade 3 GI toxicity was reported in 0.7% and 2.7% of patients, respectively. Late-grade 3 GU complications included urinary obstruction, hematuria, and fistula formation, whereas late grade 3 GI events comprised diarrhea, fecal urgency, incontinence, pain, and fistula. Overall, these findings suggest that both schedules are associated with a comparable and acceptable safety profile in the salvage setting, characterized by manageable acute effects and a low incidence of late high-grade toxicity (Fig. 3).

**Figure 3:**
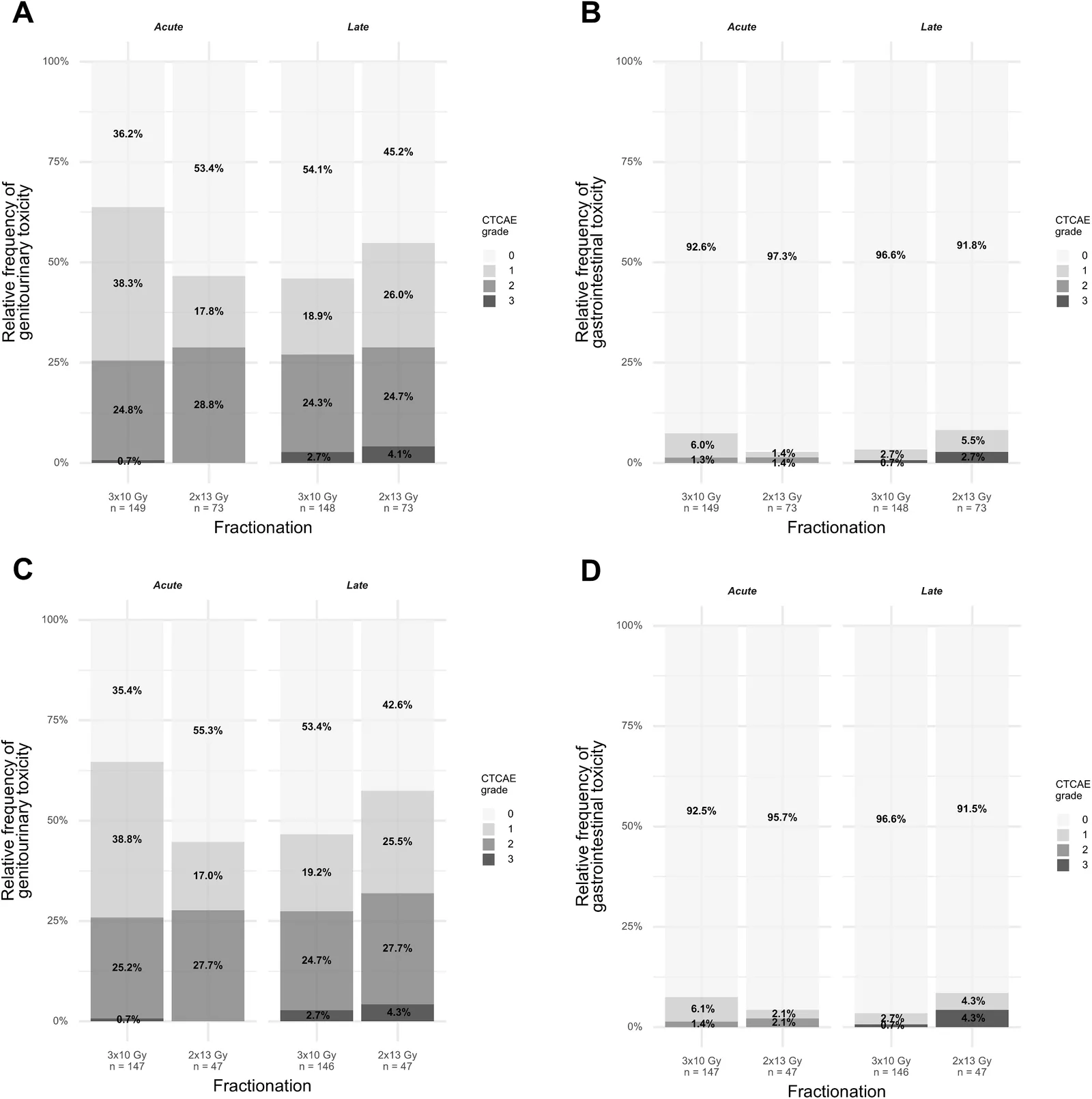
Comparison of relative frequencies of treatment toxicity between the 2 fractionation schedules. (A) Genitourinary toxicity across the entire cohort. (B) Gastrointestinal toxicity across the entire cohort. (C) Genitourinary toxicity in the subgroup with local recurrence only (N0M0). (D) Gastrointestinal toxicity in the subgroup with local recurrence only (N0M0). *Abbreviation*: CTCAE = National Cancer Institute Common Terminology Criteria for Adverse Events.

## Discussion

Our study suggests that the 2 fractionation schedules (3 × 10 Gy and 2 × 13 Gy) in sHDR-BT yield comparable outcomes, provided patient- and disease-related characteristics are taken into account. No significant survival differences in oncological outcomes were observed between the 2 dosing schedules, and, in both univariable and multivariable analysis, fractionation was not an independent predictor of any oncological endpoint in patients with local recurrence only. Both regimens achieved excellent LC and durable BC, consistent with previously reported efficacy of salvage BT.^16^^,^^20^^,^^21^ Disease extent and biology proved to be more important prognostic factors. In the whole cohort, distant metastases at recurrence were associated with a nearly 4-fold increase in risk of disease progression, whereas nodal involvement also predicted increased local failure. Castration-resistant status emerged as a consistent adverse factor, even in N0M0 patients, raising the risk of recurrence across most endpoints by approximately 2.5- to 3.8-fold. These findings are consistent with previous reports showing that hormone-refractory disease gains little benefit from local salvage treatment. Lewin et al^22^ demonstrated that 3-year recurrence-free survival after salvage reirradiation was only 9% in patients with CRPC compared with 79% in hormone-sensitive cases (*P* < .001), with a significantly higher risk of metastatic progression (*P* < .01). These findings call into question the role of salvage BT in patients with metastatic or castration-resistant recurrence, for whom systemic therapy remains the more appropriate strategy. Although toxicity was generally low, grade ≥3 complications, including rare fistulas, did occur. For patients unlikely to achieve oncological benefit, even this modest risk of morbidity may not be justified.

The subgroup of patients treated with sHDR-BT without ADT achieved encouraging outcomes, underscoring the potential of local therapy to defer or even avoid systemic treatment. In this cohort, median OS was 10 years, with median ADT-free survival of 7 years, highlighting a meaningful QoL benefit by postponing the side effects of hormonal therapy. These results are consistent with previous reports, such as that of Watanabe et al,^23^ who demonstrated that sHDR-BT alone can provide durable disease control while delaying the need for ADT in locally recurrent PCa. It should be acknowledged that the ADT-free subgroup in our study was relatively small (n = 83) and comprised generally lower-risk recurrences, which likely contributed to the favorable outcomes. Because of the limited number of events, multivariable modeling identified a few independent prognostic factors. However, in patients with N1 recurrence, a markedly higher risk of treatment failure was observed. These findings reinforce that sHDR-BT is most effective in patients with truly localized, noncastration-resistant disease. Nonetheless, the excellent survival outcomes and extended ADT-free interval in this subgroup are encouraging. They suggest that, in carefully selected patients, sHDR-BT alone can provide long-term disease control while significantly delaying systemic therapy and its toxicity.

Our results confirm that sHDR-BT can be safely delivered in experienced centers, with a low risk of serious long-term urinary or GI toxicity. This aligns with the MASTER meta-analysis, which found that sHDR-BT was associated with beneficial oncological outcomes and a favorable safety profile.^14^ Importantly, the similar efficacy of the 2 fractionation schedules after adjustment suggests that regimen choice may be guided by logistical considerations or patient preference. Ultimately, patient selection is the key determinant of success. The best candidates are those with local recurrence only, limited tumor burden, and noncastration-resistant disease. In these patients, sHDR-BT can achieve durable control and significantly delay the need for ADT, thereby preserving QoL. Conversely, patients with biologically advanced recurrence—such as rapid relapse, high PSA, ISUP grade group 5 tumors, nodal or distant metastases, or castration-resistant status—are unlikely to benefit, as treatment failure remains common despite salvage BT. For these individuals, both our data and prior studies suggest that systemic therapy should remain the cornerstone of management.^22^

Our study has several limitations inherent to its retrospective design. The treatment groups differed in several baseline characteristics, including primary treatment modalities, cumulative BED, and the interval from ADT initiation to sHDR-BT, resulting in treatment heterogeneity. In addition, follow-up was significantly shorter in the 2 × 13 Gy cohort, potentially limiting the detection of late recurrences and treatment-related toxicities. Therefore, comparisons between the fractionation schedules should be interpreted with caution. Longer follow-up and prospective validation are warranted to confirm these findings.

## Conclusions

sHDR-BT is a safe and effective treatment option for men with localized, radiorecurrent, and non-CRPC. Durable disease control and low rates of severe toxicity were observed in both fractionation regimens (3 × 10 Gy and 2 × 13 Gy). Oncological outcomes were primarily determined by disease biology. CR and regional or distant metastases were consistently associated with poorer results. These observations underscore the limited role of local salvage therapy in these settings. Importantly, in selected patients, sHDR-BT without systemic therapy provided long-term LC and BC and substantially delayed the need for ADT. Our findings reinforce that careful patient selection, rather than fractionation choice, remains the critical factor in optimizing outcomes.

## Disclosures

The authors declare that they have no known competing financial interests or personal relationships that could have appeared to influence the work reported in this paper.

## Appendix Supplementary materials

Supplementary Table S1-S2 Image, application 1

Supplementary Table S3 v2_czerw26 Image, application 2

Supplementary Table S4 v2_czerw26 Image, application 3

Supplementary Table S5 v2_czerw26 Image, application 4

## Footnotes

Supplementary material associated with this article can be found in the online version at doi:10.1016/j.adro.2026.102210.
