# Supplementary material for: Salvage High-Dose-Rate Brachytherapy for Localized Prostate Cancer Recurrence: A Comparative Analysis of 2 Fractionation Schedules—Single-Institution Study of 223 Patients
Source: Adv Radiat Oncol. 2026 Sep 17;12(1):102210. doi: 10.1016/j.adro.2026.102210 (PMC13744313; doi:10.1016/j.adro.2026.102210)
Supplement: Supplementary file 1 [file mmc1.docx]

**Table S1** Recommended and achieved dose-volume parameters for sHDR-BT.

| *Parameter* | *Recommended* | *Measure* | *Achieved* | | |
| --- | --- | --- | --- | --- | --- |
|  |  |  | *3×10 Gy*  *(n=150)* | *2×13 Gy*  *(n=73)* | *P* |
| CTV V_100_ [%] | ≥90 | *Median (Q1–Q3)* | 93.8 (90.5–95.9) | 96.4 (94.6–97.5) | <0.001 |
| CTV D_90_ [%] | ≥100 |  | 105.6 (101.0–108.7) | 109.1 (106.0–110.8) | <0.001 |
| Urethra D_10_ [%] | ≤120 |  | 118.3 (116.4–119.2) | 116.0 (115.1–117.4) | <0.001 |
| Rectum D_10_ [%] | ≤70 |  | 64.1 (58.0–67.5) | 66.8 (62.7–70.4) | 0.0007 |
| Bladder D_10_ [%] | ≤70 |  | 53.2 (46.7–59.9) | 49.1 (43.0–56.1) | 0.003 |

Abbreviations: CTV – clinical target volume, D_10_ – minimum dose received by 10% of the structure volume, D_90_ – minimum dose received by 90% of the target volume, Q1, Q3 – first and third quartiles, sHDR-BT – salvage high-dose-rate brachytherapy, V_100_ – percentage of the target volume receiving 100% of the prescribed dose.

**Table S2** Biologically effective dose (BED_1.5 Gy_) delivered to the clinical target volume according to fractionation schedule.

| *Group* | *Measure* | *BED_1.5 Gy_ – primary treatment [Gy]* | *BED_1.5 Gy_ – sum [Gy]* | *P* |
| --- | --- | --- | --- | --- |
| 3×10 Gy | *Median (Q1–Q3)* | 177.3 (172.7–202.7) | 407.3 (402.7–432.7) | 0.001 |
| 2×13 Gy |  | 182.0 (177.3–211.5) | 433.3 (428.6–462.) | <0.001 |

Abbreviations: BED – biologically effective dose, Q1, Q3 – first and third quartiles.
